# Sweet Bee Venom Triggers Multiple Cell Death Pathways or Spurs Acute Cell Rupture According to Its Concentration in THP-1 Monocytic Leukemia Cells

**DOI:** 10.3390/genes13020223

**Published:** 2022-01-25

**Authors:** Jae-Min Ryu, Han-Heom Na, Yoon-Jong Park, Jin-Sung Park, Byung-Soo Ahn, Keun-Cheol Kim

**Affiliations:** 1 Department of Biological Sciences, College of Natural Sciences, Kangwon National University, Chuncheon 24341, Korea; wkzkaos@kangwon.ac.kr (J.-M.R.); hanhum01@kangwon.ac.kr (H.-H.N.); dbswhd1598@kangwon.ac.kr (Y.-J.P.); 2 Kangwon Center for System Imaging, Kangwon National University, Chuncheon 24341, Korea; 3 AJ Research Institute for Integrative Medicine, Seoul 07525, Korea; omd9339@hotmail.com (J.-S.P.); absvision@empas.com (B.-S.A.)

**Keywords:** sweet bee venom (sBV), THP-1 cells, apoptosis, necroptosis, cell rupture

## Abstract

Sweet bee venom (sBV) contains various pharmacologically active components of bee venom (BV), but it is modified via the removal of the harmful substances found in BV. Thus, sBV has been used for pain relief in Oriental medicine but has only recently been applied for the treatment of various diseases. In this study, we examined the pharmacological effects and immunomodulatory functions of sBV in THP-1 monocytic leukemia cells. Growth inhibition and cell death were observed according to the concentration of sBV. However, the rapid collapse of cell cycle distribution was shown at 20 μg/mL sBV treatment, indicating that sBV led to cell death or acute cell rupture according to concentration. sBV administration activated Caspase-9, PARP1, RIPK1, and RIPK3, suggesting that the pharmacological actions of sBV were associated with induction of apoptosis and necroptosis. On the other hand, sBV or LPS administration increased cytokine expression, including IL-1β, and showed synergistic cell death in combinatory treatment conditions. Moreover, combinatory administration of sBV and LPS induced severe damage or death during egg development. This result implies that sBV exhibits both pharmacological and toxic effects depending on its concentration. Therefore, sBV might be a promising therapeutic approach, but optimal concentration should be considered before treatment.

## 1. Introduction

Bee venom (BV) is a well-known crude extract derived from stingers of *Apis mellifera* and contains various pharmacologically active components, including melittin, apamin, phospholipase A2 (PLA2), hyaluronidase, histamine and epinephrine [1,2]. Among these, melittin is counted as a major component (40–50%) of bee venom, followed by PLA2 (10–12%), and apamin (2–3%), respectively [3,4].

Traditionally, BV has been used to treat pain relief as a form of acupuncture in Oriental medicine [5]. BV acupuncture, remarkably, has shown antihyperalgesic effects in spinal cord dysfunction. This treatment has been assumed to involve activated spinal alpha2-adrenoceptors [6]. In addition, BV acupuncture alleviates acute cold and mechanical allodynia induced by oxaliplatin administration in A-fiber dorsal root ganglia neuronal cells, suggesting that membrane action potential might be changed by BV treatment [7]. Recently, therapeutic BV has also been applied as a possible treatment for inflammatory diseases [8,9,10]. BV suppressed NO production and decreased the levels of iNOS, COX-2, NF-κB and MAPK in RAW264.7 macrophages, suggesting that BV has an anti-inflammatory effect [11]. BV acupuncture significantly alleviated ear skin symptom severity and thickness, inflammation, and lymph node weight in trimellitic anhydride (TMA)-induced atopic dermatitis mouse models and inhibited the expression levels of both T helper cell type 1 (Th1) and Th2 cytokines at the molecular level [12]. Mellitin, a major component of BV composed of 26 amino acids, reduced paw edema and nociceptive behaviors in the injected side of the paw via decreased Fos expression in the superficial layer of the lumbar spinal cord [13]. PLA2 decreased lipid accumulation in the liver and reduced kidney inflammation in high-fat diet mice through immunomodulatory effects on macrophages, suggesting that bvPLA2 could be a potential therapeutic candidate on obesity by regulating macrophage homeostasis in adipose tissue [14].

However, another important concern during BV treatment is acute induction of anaphylaxis [15]. A case report showed that a patient with BV acupuncture experienced intravascular coagulation, and finally death by hypovolemic shock, even though the victim had no history of any medical disorders, reaction to bee stings, or allergies [16]. A cohort study showed that the median frequency of adverse reactions was 28.87% of patients who experienced venom immunotherapy, suggesting that BV therapy should be cautiously applied, considering quality and quantity [17]. As part of various efforts, sweet bee venom (sBV) has been developed, removing the harmful substances of BV that could trigger anaphylaxis [18]. A case report suggested that sBV treatment showed more effective results than BV treatment in controlling itching but not in controlling pain [19].

For this reason, sBV might show promising pharmacological efficacy on various disease models compared to BV. Although sBV has been suggested as a safer product than BV, further studies are required to clarify the molecular mechanism of sBV administration. Specifically, a scientific investigation should be introduced on the safety and effectiveness of sBV to utilize as a novel therapeutic approach. Therefore, we performed these experiments to examine the pharmacological effects and immunomodulatory functions of sBV in THP-1 monocytic leukemia cells. Interestingly, we found that sBV administration showed double-edged effects on the induction of cell death or cell rupture according to its concentration or combinatory treatment condition with LPS.

## 2. Materials and Methods

### 2.1. Cell Culture and Reagents

THP-1 cells were obtained from ATCC (USA) and maintained in RPMI 1640 medium supplemented with 10% fetal bovine serum and 1% penicillin/streptomycin. Sweet bee venom (sBV) was kindly provided by the Korean Society of Acupuncture. The sBV was dissolved in PBS (50 μg/mL) and kept at −20 °C. Lipopolysaccharides (LPS) were purchased from Sigma (USA) and dissolved in PBS (1 μg/mL), and aliquots were stored in −20 °C.

### 2.2. Cell Viability Assay

The sBV was treated in culturing THP-1 cells in a dose-dependent manner, and then cells were counted with 0.4% Trypan blue staining solution (Thermo Fisher, Waltham, MA, USA). Living cells and dead cells were observed with a phase-contrast microscope. In total, 1 × 10^4^ cells were seeded in 96-well plates for MTT assay. The sBV was administrated in each well in triplicate and incubated for up to 48 h. Cell viability was determined by incubation with 0.5 mg/mL MTT solution (Sigma Aldrich, Saint Louis, MO, USA) for 4 h. The amount of MTT-formazan was determined at 570 nm absorbance. The ATP level was measured with a molecular probe ATP determination kit using a luminometer (Thermo Fisher, Waltham, MA, USA). All the reagents were prepared as recommended by the manufacturer’s protocol. Luciferase activity was obtained on a luminometric reader (BioTek, Winooski, VT, USA). The procedure was repeated at least 3 times. 

### 2.3. FACS Analysis

The THP-1 cells were cultured after treatment with various concentrations of sBV. The cells were harvested, washed twice in PBS, and fixed in 75% ethanol for 24 h. Cells were stained with propidium iodide (50 mg/mL) for 30 min. Cell cycle distribution was analyzed with flow cytometric analysis according to Becton Dickinson’s protocols.

### 2.4. Western Blot Analysis

Proteins were extracted using RIPA lysis buffer (25 mM Tris-HCl pH 7.6, 150 mM NaCl, 1% NP-40, 1% sodium deoxycholate, 0.1% SDS) and supplemented with proteasome inhibitors. Protein concentrations were measured using Bradford reagents (Thermo Fisher, Waltham, MA, USA). A total of 50 μg of protein was separated by SDS-PAGE gel electrophoresis and transferred into polyvinylidene fluoride (PVDF) membranes. The membranes were incubated with specific primary antibodies, followed by horseradish peroxidase-conjugated secondary antibodies (Santa Cruz, Dallas, TX, USA). Finally, the proteins were detected using an ECL protein detection kit (Amersham Inc., Amersham, Buckinghamshire, UK). Primary antibodies for caspase 9, phospho-RIPK1, RIPK1, RIPK3, and IL-1β were purchased from CST (Danvers, MA, USA). PARP1, BAX, BCL2, Cyclin D3, Cyclin A, Cyclin B, and β-actin were obtained from Santa Cruz (Dallas, TX, USA). 

### 2.5. RT-PCR

Total RNA was isolated using the TRIzol reagent method (Thermo Fisher, Waltham, MA, USA). cDNA was synthesized using 1 μg of total RNA with M-MLV reverse transcriptase (Invitrogen, Waltham, MA, USA), and then followed by PCR amplification with appropriate primer sets. Taq polymerase was purchased from Biosolution, Inc. (Suwon, Kyunggi, Korea). Primer sets were described by the following: IL-1α (sense; 5′-TCGCCAATGACTCAGAGGAA-3′ and antisense; 5′-TGGTCTTCATCTTGGGCAGT-3′), IL-1β (sense; 5′-TCCAGCTACGAATCTCCGAC-3′ and antisense; 5′-TGGAGGTGGAGAGCTTTCAG-3′), TNF-α (sense; 5′-GAGTGACAAGCCTGTAGCCCATGTTGTAGC-3′ and antisense; 5′-GCAATGCCAAAGTAGCCTGCCCAGAC-3′), and β-actin (sense; 5′-GGATTCCTATGTGGGCGACGA-3′ and antisense; 5′-CGCTCGGTGAGGATCTTCATG-3′). Agarose gel electrophoresis was performed to separate the PCR products.

### 2.6. Immunostaining

The cells were grown on coverslips for the immunostaining experiments. The coverslips were fixed in 4% paraformaldehyde for 10 min, permeabilized in 0.1% Triton X-100 for 10 min and blocked in 5% BSA for 1 h. Cells were incubated with primary antibody overnight at 4 °C, and then followed by incubation with Alexa Fluor 488 or Alexa Fluor A555-conjugated secondary antibodies (Thermo Fisher, Waltham, MA, USA). A 4′, 6-diamidino-2-phenylindole (DAPI) was used for nuclear staining. Finally, the coverslips were mounted onto glass slides with anti-fade reagent, and the fluorescence images were observed using a Ti2 confocal microscope from the Kangwon Center for System Imaging (Nikon, Minato, Tokyo, Japan).

### 2.7. Egg Development and Blood Vessel Counting

Brown Leghorn hen’s eggs were purchased from local farms near Chuncheon, Korea. The eggs were horizontally placed in a 37 °C incubator to allow exposure of the chick chorioallantoic membrane (CAM) by detaching the membrane from the eggshell (Day 0). On day 3 of incubation, the eggshell was opened with sterilized scissors, and the CAM was exposed. On day 8 of incubation, sBV or LPS was administrated on the CAM membrane after a mixture with matrigel agarose as a scaffold (Corning Inc., Corning, NY, USA). The eggshell window was sealed with a paraffin film to maintain sterilization and was examined upon egg development thereafter. On day 18 of incubation, blood vessel formation was monitored and measured by ImageJ software (version. 1.53).

### 2.8. Statistical Analysis

All statistical analyses were performed with GraphPad Prism software (GraphPad Software, La Jolla, CA, USA). All values for the experiments are presented as means ± SD (standard deviation). The number of biologically independent samples was used in a triplicated manner. The statistically significant difference between groups was assessed by a Student’s *t*-test. Statistical significance was indicated by n.s.—no significance, * *p* < 0.1, ** *p* < 0.05, *** *p* < 0.01, **** *p* < 0.001

## 3. Results

### 3.1. sBV Administration Shows Growth Inhibition or Cell Rupture According to Concentration

**Figure 1:**
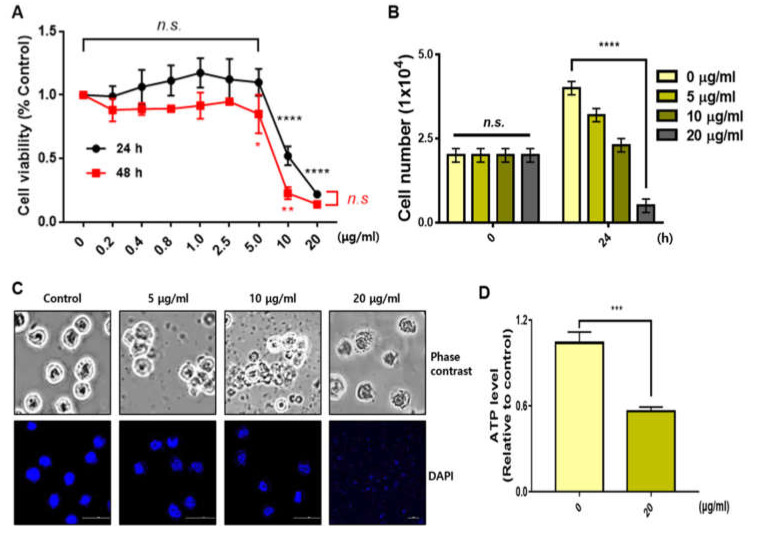
Distinct cell death effect depending on sBV concentration. (**A**) THP-1 cells treated with various concentrations of sBV for 24 or 48 h. A total of 10 μg/mL of sBV or more shows the growth inhibitory effect. (**B**) THP-1 cells counted after staining with trypan blue solution. (**C**) Morphology of cell death detected in a dose-dependent manner. Cell rupture observed in 20 μg/mL concentration. (**D**) ATP level was measured using a luminometer after 1 h treatment with 20 μg/mL of sBV. Data are presented as mean ± SD. n.s.—no significance, * *p* < 0.1, ** *p* < 0.05, *** *p* < 0.01, **** *p* < 0.001 (Student’s *t*-test).

We treated various concentrations of sBV in THP-1 cells and performed an MTT assay after 24 or 48 h. The viability of the cells was slightly decreased in concentrations below 2.5 μg/mL of sBV, but was severely decreased in concentrations above 5 μg/mL of sBV (Figure 1A). Cell proliferation by sBV was also confirmed by cell counting. The sBV administration showed decreased cell numbers in a dose-dependent manner, but it was almost impossible to observe living cells at 20 μg/mL of sBV treatment (Figure 1B). Microscopic analysis showed that the decreased cell number and dead cells were observed according to the concentration of sBV. However, interestingly, we also observed that the cells burst out at concentrations of 20 μg/mL.

**Figure 2:**
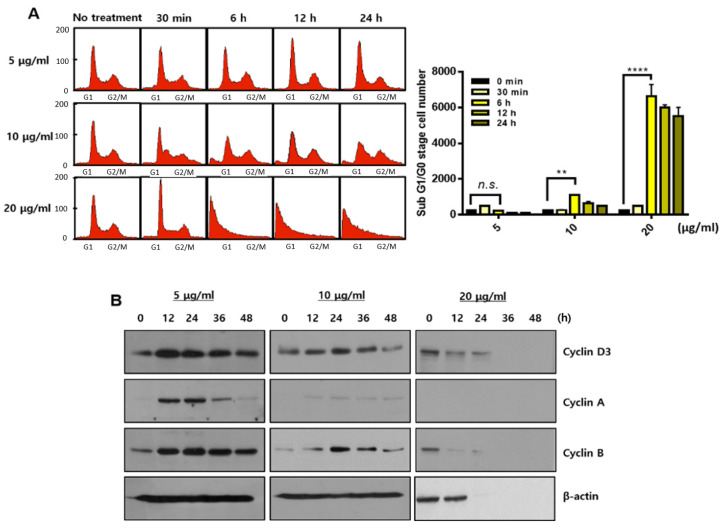
Changes in cell cycle distribution depending on sBV concentration. (**A**) FACS analysis performed after sBV treatment and calculated on sub-G1 cells. (**B**) THP-1 cells treated with the indicated concentration of sBV and western blot analysis performed using cyclin antibodies. Data are presented as mean ± SD. n.s.—no significance, ** *p* < 0.05, **** *p* < 0.001 (Student’s *t*-test).

There were no stained nuclei in the 20 μg/mL concentration in the DAPI staining experiment, indicating that high concentrations of sBV induced acute cell lysis or rupture. We also measured ATP levels to ascertain the net impact on energy production. The ATP level was severely decreased by 1 h of treatment with 20 μg/mL of sBV, indicating that a high concentration of sBV shows acute changes in metabolic activity. FACS experiments were performed to analyze the amount of apoptosis or the amount of cell cycle phase. THP-1 cells (0 min) on the left were collected before sBV treatment and used as negative control groups (Figure 2A). In addition, we performed non-treated negative control experiments for every hour of the sBV administration. All negative FACS data were similar to the 0 min cell cycle distribution. Cell cycle alterations were also examined with FACS analysis after sBV treatment. G1 arrest slightly increased from 12 h treatment of 5 μg/mL sBV, and the increase in the number of sub-G1 cells was detected after 6 h administration of 10 μg/mL sBV. However, rapid G1 arrest was shown at 30 min after 20 μg/mL sBV treatment, followed by the rapid collapse of cell cycle distribution. Quantitatively, the population of sub-G1 staged cells was dramatically increased in the 20 μg/mL sBV-treated groups after 6 h. Western blot analysis suggested that each cyclin expression was detected up to 10 μg/mL of sBV, but the cyclin proteins were not detected in 20 μg/mL sBV treatment (Figure 2B). sBV administration at a concentration of 5 μg/mL slightly increased the expression of cyclin D3. In addition, it was observed that the expression of cyclin A and cyclin B changed with the 5 μg/mL treatment of sBV. The increased expression of Cyclin A might have been associated with the increased G1 population, as shown in FACS analysis. However, the expression of cyclin A proteins quantitatively decreased in the treatment with 10 μg/mL of sBV, suggesting that the cell cycle arrest was converted into a cell death mechanism in this concentration. In particular, the expression of most cyclin proteins could not be detected at a 20 μg/mL concentration, indicating that the THP-1 cells were completely destroyed. Together, these data suggest that sBV might have pharmacological activity of cell death induction after growth inhibition or acute cell rupture according to concentration. 

### 3.2. Pharmacological Action of sBV Is Associated with Induction of Apoptosis and Necroptosis 

**Figure 3:**
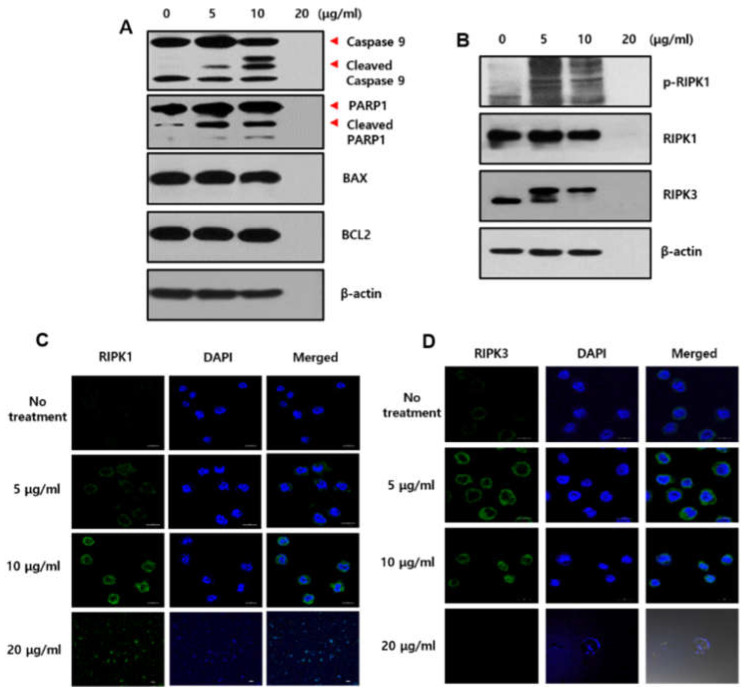
Changes in cell death marker proteins in sBV-treated THP-1 cells. (**A**) THP-1 cells treated with the indicated concentration of sBV for 24 h, and Western blot analysis performed on apoptosis marker proteins including Caspase 9, PARP1, BAX, and BCL2. (**B**) Western blot analysis performed on necroptosis marker proteins RIPK1 and RIPK3. (**C**). Immunostaining experiment performed with RIPK1 antibody. (**D**) Immunostaining experiment was performed with RIPK3 antibody.

We performed a Western blot analysis to examine which molecular mechanism was involved in sBV-treated THP-1 cells. Apoptosis marker proteins, including cleaved-Caspase 9 and cleaved PARP1, increased by ~10 μg/mL sBV treatment but were not detected in 20 μg/mL sBV treatment (Figure 3A). The BAX or BCL2 protein was not changed in this experiment. A Western blot analysis on necroptosis marker proteins was also performed. The expression of the total RIPK1 protein was not changed, but the phosphorylated form of RIPK1 was increased by sBV treatment. On the other hand, the RIPK3 protein is normally detected in 55 kDa but was detected in 60 kDa by sBV treatment, suggesting that necroptotic cell death also was caused by sBV treatment (Figure 3B). The expression of RIPK1 and RIPK3 proteins was also observed during the immunostaining experiment. RIPK1 protein was slightly increased in cytoplasmic regions. Although there was no quantitative change in total RIPK1 except for the increase in p-RIPK1 in the Western blot experiment, these immunostaining data could be considered as an increased pattern of activated RIPK1. The RIPK3 protein was localized near the nuclear membrane in ~10 μg/mL sBV treatment, suggesting that the RIKP3 protein was activated by sBV treatment (Figure 3C,D).

### 3.3. Combinatory Treatment of sBV and LPS Shows Synergistic Cell Death via Cytokine Expression

**Figure 4:**
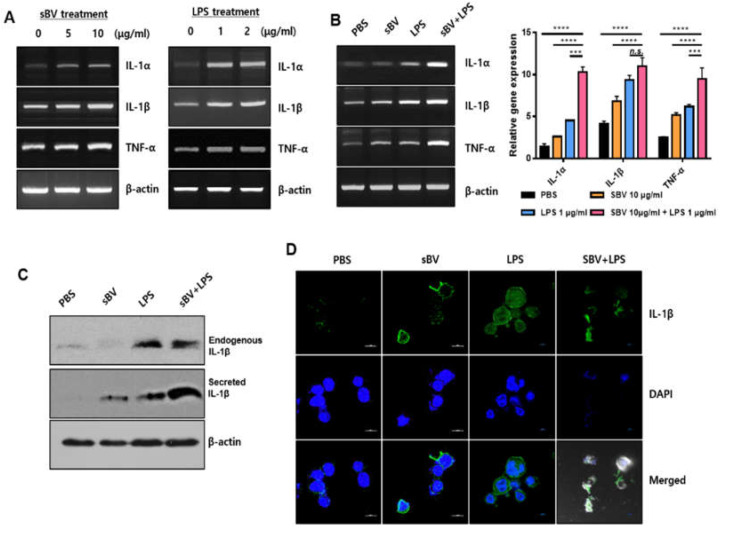
Synergistic cytokine expression in combinatory treatment with sBV and LPS. (**A**) sBV (left) or LPS (right) treated in THP-1 cells, respectively. RT-PCR performed to examine cytokine expression including IL-1α, IL-1β, and TNF-α. (**B**) RT-PCR performed after combinatory treatment of sBV and LPS. (**C**) Cytoplasmic or secreted IL-β protein evaluated using Western blot analysis. (**D**) Immunostaining experiment performed with IL-β antibody. Data are presented as mean ± SD. n.s.—no significance, *** *p* < 0.01, **** *p* < 0.001.

**Figure 5:**
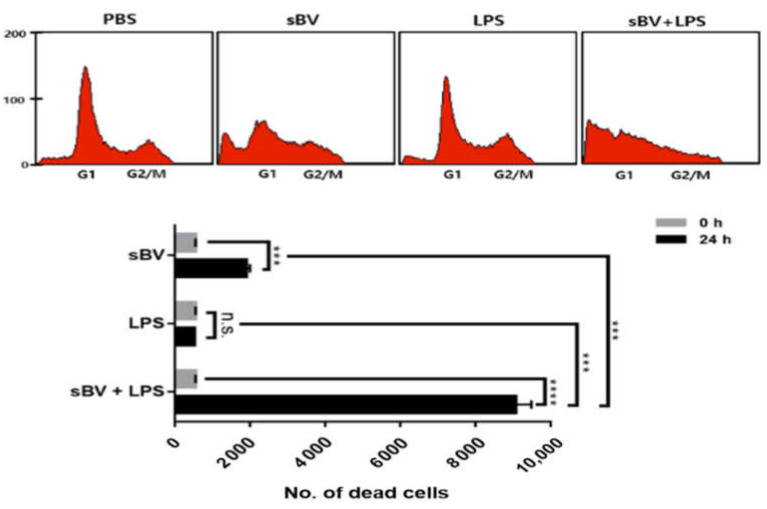
Synergistic cell death effects in combinatory administration with sBV and LPS. FACS analysis performed to examine synergistic effect of sBV and LPS in THP-1 cells. Data are presented as mean ± SD. n.s.—no significance, *** *p* < 0.01, **** *p* < 0.001.

We examined inflammatory cytokine expression after sBV administration in THP-1 cells. The RT-PCR analysis showed that sBV treatment upregulated IL-1α, IL-1β and TNF-α in a dose-dependent manner (Figure 4A). Lipopolysaccharide (LPS), an inducible endotoxin of the immune response, also upregulated IL-1α, IL-1β and TNF-α of THP-1 cells. These cytokine genes were synergistically increased in the combinatory treatment of sBV and LPS, indicating that the pro-inflammatory cytokines were tightly regulated by the administration of sBV and LPS (Figure 4B). We examined amounts of intracellular and secreted forms of IL-1β in the combinatory treatment using Western blot analysis (Figure 4C). The IL-1β protein was increased as cytoplasm or in a secreted form by sBV or LPS treatment and was detected in higher amounts in combinatory treatment of sBV and LPS. These data suggest that IL-1β proteins were increased in sBV and LPS stimulation via transcriptional regulation. Immunostaining on IL-1β showed that the protein was mainly detected at the perinuclear region by sBV administration, whereas it was shown as a dotted pattern in LPS-treated cells (Figure 4D). Interestingly, we could not detect typical DAPI staining patterns in the combinatory administration of sBV and LPS, although IL-1β may be detected in its secreted form. The increased dead cells were detected in the combinatory treatment of sBV and LPS using FACS analysis, indicating that the combinatory administration of sBV and LPS was associated with the induction of strong cell death (Figure 5). These data suggest that sBV or LPS treatment increased cytokine expression, including IL-1β, and showed synergistic cell death in combinatory treatment conditions.

### 3.4. Egg Development Was Stopped

**Figure 6:**
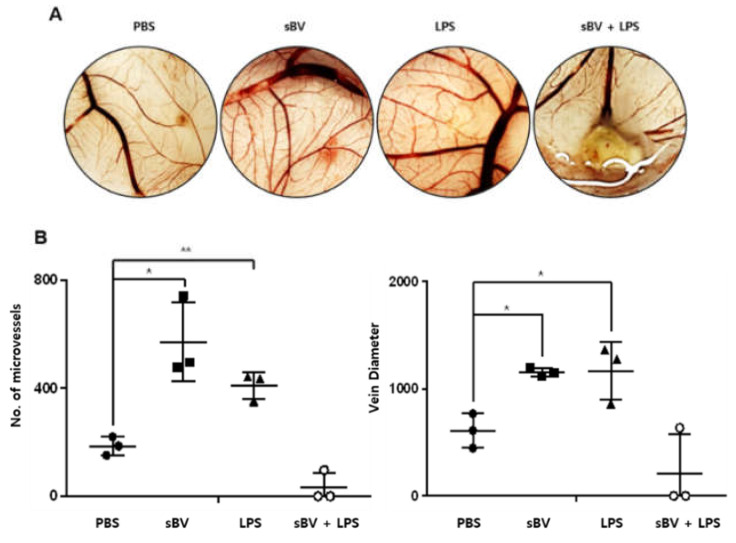
Failure of egg development by combinatory administration of sBV and LPS. (**A**) sBV and LPS mixed with matrigel and administrated on chorioallantoic membrane (CAM) during egg development. Normal development was maintained in sBV- or LPS-treated groups, but failed in combinatory administration groups. (**B**) Calculations of microvessel formation and vein diameters. Data are presented as mean ± SD. * *p* < 0.1, ** *p* < 0.05.

To examine the synergistic effects of the combinatory treatment of sBV and LPS, we treated sBV and LPS on the chorioallantoic membrane (CAM) at day 8 of egg development. Egg development was normally progressed in the non-treated group and was also shown in the treatment groups of sBV or LPS (Figure 6A). However, combinatory treatment of sBV and LPS induced severe damage to death in failing development from 4–5 days after cotreatment. We also performed a quantitative analysis of the blood vessel by measuring vein diameter and microvessel formation. Administration of sBV or LPS increased the number of microvascular (branches) and the diameter of the vein compared with the control group (Figure 6B). However, these calculations could not be performed in the combinatory treatment of sBV and LPS due to the failure of egg development. These data strongly suggest that combinatory treatment of sBV and LPS has synergistic activity in inhibiting normal development of the egg.

## 4. Discussion

Therapeutic approaches using BV have been tried in various forms, including direct administration of live bee stings and BV acupuncture [20,21]. Despite the successful clinical outcome of BV as a pharmacological drug, it has also been shown to have unexpected side effects such as anaphylaxis [22]. Anaphylaxis by BV has been reported by several papers, but actual cases might be far more numerous [16,23]. Since anaphylaxis is a severe immunological reaction that can occur within seconds or minutes of exposure to allergens, THP-1 human monocytic leukemia cells might be appropriate in vitro model cells for studying hypersensitive reactions [24,25,26]. 

In this study, we showed distinct pharmacological efficacies on cell death patterns depending on the concentration in sBV-treated THP-1 cells. In concentrations below 10 μg/mL of sBV, THP-1 cells showed increased cell death via growth inhibition in dose-dependent manners. However, THP-1 cells showed acute cell rupture or cell lysis in high concentrations (20 μg/mL) of sBV, indicating that sBV had different effects depending on its concentration. Typical cell death marker proteins were detected in the treatment concentrations below 10 μg/mL of sBV. In this range of concentration of sBV, cleaved forms of Caspase 9 and cleaved PARP1 increased. Activated Caspase 9 initiated apoptotic cell death by sequential activation of downstream executioner caspases and finally induced PARP cleavage in cell nuclei, indicating that sequential activation by sBV induced apoptosis of THP-1 cells [27,28]. Necroptotic proteins RIPK1 and RIPK3 were also activated by sBV treatment in our study. Necroptosis is a type of programmed cell death with necrotic morphology, occurring in a variety of biological processes, including inflammation, immune response, embryonic development and metabolic abnormalities [29,30,31]. RIPK1/RIPK3 has been known as a critical regulator of necroptosis and inflammation. RIPK1/RIPK3 is activated by phosphorylation, or Caspase-mediated cleavage [32,33]. These post-translational modifications, in coordination, regulate the assembly of a macromolecular signaling complex [34]. Therefore, it was hypothesized that the multiple cell death mechanism proceeds in the direction of strong mutual regulation for the enhanced pharmacological effects during sBV treatment. These results suggested that pharmacological concentrations of sBV induced complex cell death mechanism in THP-1 leukemia cells. The involvement of the NF-kB signaling pathway in the apoptotic cell death by BV and melittin was also shown in various human cancer cells [35,36]. Therefore, the induction of multiple cell deaths by sBV might be involved in the NF-kB pathway, but this needs to be clarified in THP-1 cells.

On the other hand, our current study showed that several cytokines, including IL-1α, IL-1β, and TNF-α, were produced within pharmacological concentrations of sBV. Moreover, the expression of these pro-inflammatory cytokines was increased by combinatory treatment of sBV and LPS. Although inflammatory cytokines are key mediators of the inflammatory response within tumor cells, the functional role of cytokine expression in cancer cells is controversial [37,38]. IL-1β had a significantly pro-apoptotic effect under various conditions [39]. Production of the inflammatory cytokine IL-1β requires two distinct signals: first, a signal that induces de novo pro-IL-1 β gene transcription through NF-kB and a second, inflammasome-dependent signal that cleaves pro-IL-1β to produce the mature cytokine [40,41]. Moreover, TNF-α is not only a pleiotropic cytokine in inflammation, but it also exhibits effects such as apoptosis through death receptors containing a death domain [42]. TNF-α triggers either NF-κB activation or RIPK1 kinase-dependent cell death [43]. Therefore, the increase of TNF-α by sBV might be related to the enhancement of the necroptosis-related mechanism. Several previous papers have suggested that cytokines could inhibit cancer cell survival development and progression [44,45]. Alternatively, cancer cells can respond to host-derived cytokines that promote growth, attenuate apoptosis and facilitate invasion and metastasis [46,47,48].

Interestingly, the increased dead cells were detected in the combinatory treatment of sBV and LPS using FACS analysis, indicating that the combinatory administration of sBV and LPS was associated with the induction of strong cell death. Most IL-1β proteins might be secreted to extracellular regions, suggesting that inflammasome activation of caspases might be associated with the mature form of IL-1β in sBV-treated THP-1 cells [49,50,51]. We also observed similar phenomena in this experiment through CAM assay. Vessel formation was increased by treatment with sBV or LPS alone but was not normally developed in the combinatory treatment of sBV and LPS. The chick chorioallantoic membrane (CAM) is an extra-embryonic membrane, comprised of a high density of blood and lymphatic vessels [52]. The CAM has a dense capillary network and is commonly used to study in vivo angiogenesis and anti-angiogenesis in response to potential biomolecules and drugs [53]. Therefore, these results prove that the combination of sBV and LPS can exert a strong apoptotic effect.

Our current experimental results show that the pharmacological effects of sBV were converted into rapid cell rupture at a concentration of approximately 20 μg/mL in THP-1 cells. In addition, acute cell rupture was observed at a similar concentration in sBV-treated A549 lung cancer cells. Therefore, we suggest that concentration below 10 μg/mL of sBV shows pharmacological effects in most cell-based experiments, but concentrations of 20 μg/mL and more show side effects. Future experiments are required to determine the effective pharmacological concentration in mouse models. Moreover, the pharmacological concentration of sBV should be considered in clinical trials, if it is possible. According to the previous literature, the effects of drugs and poisons were determined to be closely related to the concentration applied [54,55,56]. Thus, we can suggest that sBV exhibits both pharmacological effects and toxic effects depending on its concentration. However, once the anaphylaxis caused by BV is considered as an unexpected phenomenon, like cell rupture, we can predict the concentration of side effects caused by sBV. sBV shows cell death or anaphylaxis depending on the concentration applied. Additionally, individual differences in concentration will be an important factor for the induction of pharmacological effects or anaphylaxis. Therefore, sBV might be a promising therapeutic approach, but the optimal concentration should be considered prior to sBV administration.

## Data Availability

All data used in this paper are available in the article.
